# Infantile Hemangiomas: An Update on Pathogenesis and Treatment

**DOI:** 10.3390/jcm10204631

**Published:** 2021-10-09

**Authors:** Małgorzata Kowalska, Wojciech Dębek, Ewa Matuszczak

**Affiliations:** Department of Pediatric Surgery and Pediatric Urology, Medical University of Bialystok, Waszyngtona 17, 15-274 Bialystok, Poland; wojciech.debek@umb.edu.pl (W.D.); ewamat@tlen.pl (E.M.)

**Keywords:** infantile hemangioma, propranolol, PDL

## Abstract

Infantile hemangiomas are the most common benign vascular tumors in infancy. This review includes an update on the current knowledge on pathogenesis, a discussion on indications for treatment, and a review of the mechanisms underlying the different treatment methods. Although most infantile hemangiomas require only active observation because of their natural course, which results in involution, about 10% present with complications that require immediate treatment. The basic treatment includes systemic and topical options. In cases of insufficient response or rebound growth, other forms of treatment should be considered. In some cases, combined therapy might be initiated.

## 1. Introduction

### 1.1. Definition and Epidemiology

Infantile hemangiomas (IHs) are the most common benign vascular tumors in infancy and present in up to 5% of children [1]. In this article, we would like to review the current knowledge regarding the pathogenesis and treatment of infantile hemangiomas. Usually, IHs are not visible after birth or present as a faint discoloration with a light halo and become evident in a period of 1–2 weeks [2]. Eighty percent of IHs appear in the face and neck region. In the case of deeply localized IHs presenting as bluish tumors without clear borders, the diagnosis may be delayed until up to 3 months after birth. The natural cycle of IHs consists of three phases: the rapid proliferation phase (with the fastest growth between 5.5 and 7.5 weeks), the plateau phase, and the slow involution phase. The maximum size is achieved at around 9 months on average, and regression is completed by the age of 4 years in 90% of cases [1].

### 1.2. Risk Factors

The meta-analysis performed by Ding et al. showed that the risk factors for IH are female gender, multiple gestation, preterm birth, progesterone therapy, family history of IH, and low birth weight (every 500 g decrease in birth weight increases the risk by 40%) [3,4].

### 1.3. ISSVA 2018 Classification

According to the newest ISSVA (International Society for The Study of Vascular Anomalies) 2018 classification, IHs can be divided into different patterns: focal, multifocal, segmental, and indeterminate. These, in addition to size and location, affect the chosen treatment approach. Complications are more common among patients with segmental IH, and hence there is a greater need for therapy [5].

### 1.4. Differential Diagnosis

It is important to distinguish IHs from other vascular tumors. Congenital hemangiomas (CHs), like IHs, are caused by cellular hyperplasia but are present at birth and are caused by a mutation in the GNAQ and GNA11 genes. They subdivide into rapidly involuting CHs (RICHs), non-involuting CHs (NICHs), and partially involuting CHs (PICHs). Although some may resemble the natural cycle of IH, a lack of GLUT1 staining confirms the diagnosis of CH.

Vascular malformations are another group of vascular anomalies caused by pathological vascular morphogenesis. Usually present at birth (although not always visible), they do not show growth and spontaneous regression potential [6,7].

### 1.5. Syndromes Associated with IH

Despite their benign character, IHs may be associated with organ anomalies in some locations. PHACES syndrome consists of posterior fossa malformations, hemangioma (usually localized on the face), arterial anomalies, cardiovascular anomalies, eye anomalies, sternal clefting and/or supraumbilical raphe. Severe complications include an arterial ischemic stroke, as the most common extracutaneous manifestations are arterial anomalies of cerebral vessels and aortic arch. Another syndrome-associated location is a hemangioma of the lower body, which may suggest LUMBAR syndrome: lower body hemangioma, urogenital anomalies, ulceration, myelopathy, bone deformities, anorectal malformations, arterial anomalies, and renal anomalies [1].

Multiple cutaneous hemangiomas may also suggest extracutaneous involvement. The most frequently affected organ is the liver, with a multifocal or diffuse pattern of IH. Although they follow the natural proliferation and involution course of IH and most are asymptomatic, a few might be associated with complications such as bleeding, high-output congestive heart failure, or hypothyroidism [7,8].

### 1.6. Complications

The possible complications of infantile hemangiomas include ulceration, disfigurement, obstruction, and functional impairment. Ulceration is the most common complication and occurs in up to 25% of patients; it is associated with larger IHs and segmental hemangiomas. The high-risk areas are the head and neck, the axilla, and the anogenital region [1,9]. Disfigurement usually applies to residual lesions after natural involution or treatment and may present as fibrofatty tissue, anetoderma, scarring, telangiectasia, or deformations [10]. Obstruction and functional impairment are commonly associated with IHs located in the head and neck area and around natural orifices of the body [1,7].

## 2. Pathogenesis

Over the years, scientists have postulated what seemed to be different theories of the pathogenesis of IH. It now appears that most of them are somehow connected. In recent studies, authors have suggested that IHs are a result of dysregulated vasculogenesis (i.e., the formation of new blood vessels from stem cells) and angiogenesis (i.e., the formation of new blood vessels from existing vessels). Hypoxia, which seems to be the trigger for this dysregulation, causes the overexpression of angiogenic factors such as vascular endothelial growth factor (VEGF) by inducing the transcription of the VEGF gene. Although it is believed that the induction of transcription of the VEGF gene proceeds via the hypoxia-inducible factor-1 alpha (HIF-1-alpha) pathway, a recent study has shown no significant change in its levels compared to hypoxia-inducible factor-2 alpha (HIF-2 alpha), which is upregulated in the proliferative phase and decreases over time, making it a more likely gene-inducing factor. Prolonged hypoxic conditions downregulate the levels of HIF-1 alpha and cause the accumulation and stabilization of endothelial PAS domain protein 1 (EPAS1) mRNA, which encodes for transcription factor HIF-2 alpha. Gomez-Acevedo et al. also confirmed the suppression of the mRNA of the enzyme aldehyde dehydrogenase 1 (ALDH1A1) in the proliferation phase, which has been suggested to be lower in hypoxic conditions [11]. This evidence strongly supports the presence of hypoxic conditions in the early phase of IH development.

IH stem cells, the cellular precursors of IH, have the ability to differentiate into endothelial cells which express a unique phenotype: indoleamine 2,3-dioxygenase (IDO), LYVE-1, CCR6, glucose transporter-1 (GLUT-1), antigen Lewis-Y (Ley), antigen FcγRII, merosin, and CD15. The GLUT-1, highly expressed in human brain and placenta, and the vascular antigens expressed in the fetal microvessels of placental tissue (antigen Lewis-Y (Ley), antigen FcγRII, merosin) link the theory of placental embolization to IH pathogenesis. The origin of IH stem cells was proposed to be in placental tissue, from where they migrate and usually localize on head and neck, along the lines of fusion of the facial placodes, as “benign metastases” [12]. Another argument supporting this thesis is a recent discovery by Strub et al. They reported that the chromosome 19 miRNA cluster (C19MC), expressed in the placenta and rarely in postnatal tissues, is also expressed in IH endothelial cells. The levels of C19MC miRNAs were found to be elevated solely in the circulation of patients with IH, but neither in other vascular anomalies nor in the control group. Moreover, the circulating C19MC microRNA levels showed a correlation with the tumor size and clinical response to oral propranolol, emerging as a first potential biomarker for IH [13]. Contrary to the placental origin of IH, a recent work by Moisan et al. reported that IH endothelial cell staining was negative for Aquaporin-1 (AQP1), while endothelial cells derived from placental tissue were AQP1 positive [14,15].

Other upregulated factors contributing to vessel proliferation in proliferating hemangiomas include basic fibroblast growth factor (bFGF), vascular endothelial growth factor receptor (VEGFR), matrix metalloproteinases (MMPs), proliferating cell nuclear antigen, type IV collagenase, and components of the RAA (renin–angiotensin–aldosterone) axis [16]. It was shown that the mRNA expression of angiotensin-converting enzyme (ACE) and angiotensin II receptor type 1 (AGTR1) was elevated in all stages of IH compared to the control group. The mRNA levels of angiotensinogen (AGT) were also significantly upregulated in the proliferating phase of IH in comparison to normal skin tissue. The contribution of the RAA axis to IH pathogenesis could explain the natural course of IH involution with age. The physiological decrease of renin levels in infants over time and the reduction of renin induced by beta-1-adrenergic antagonists (e.g., propranolol) could explain the process of involution, spontaneous and accelerated by drug therapy [17], respectively. It is worth mentioning that all necessary components of the RAA system are expressed in human placental tissue [18].

Understanding the pathogenesis of IH is crucial for identifying patients at the highest risk of complications caused by the size or location of IH, and for developing a therapy with the most beneficial ratio of efficacy to adverse effects.

## 3. Treatment

**Figure 1:**
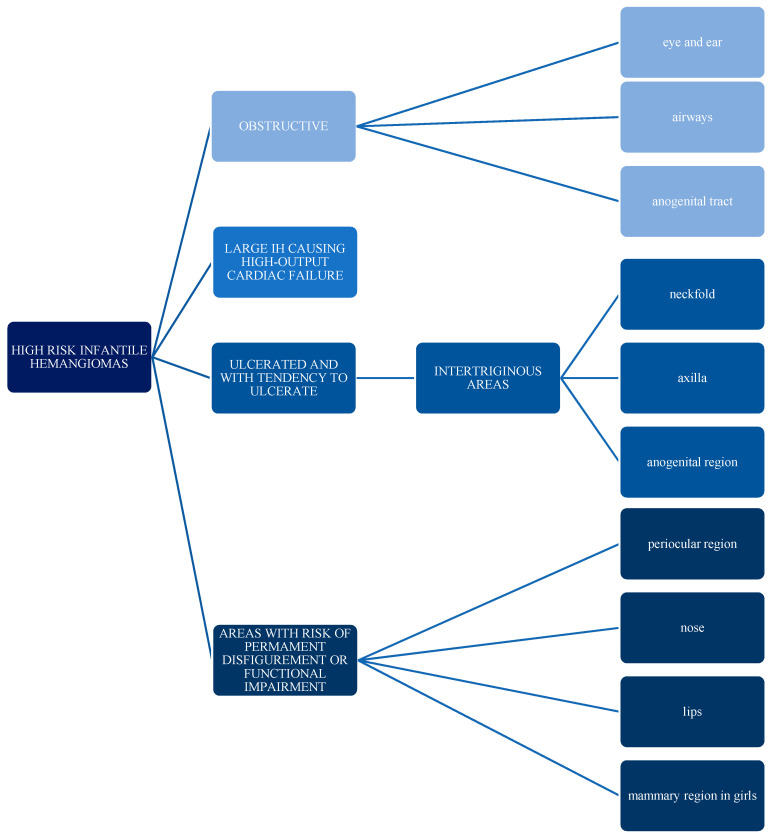
Characteristics of infantile hemangiomas with a high risk of complications.

**Figure 2:**
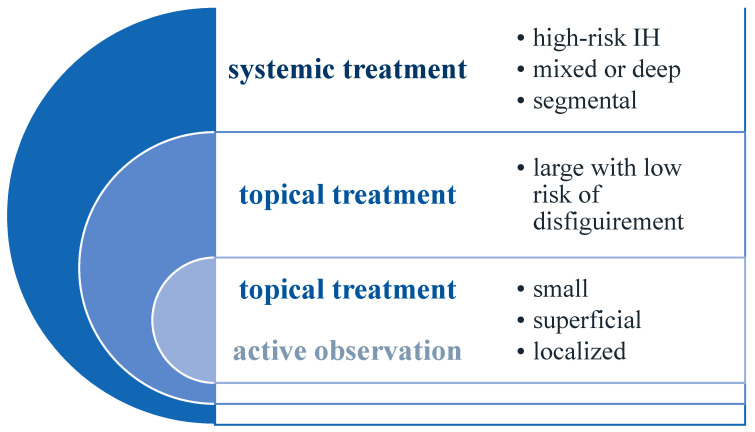
Treatment indications considering size, depth, and the morphological character of infantile hemangiomas.

Since 2008, when Leaute-Labreze et al. discovered the beneficial effects of propranolol for infantile hemangiomas, beta-blockers became the first line of treatment and ended the era of glucocorticosteroids as the gold standard [19]. Most hemangiomas do not require immediate treatment due to their self-involuting pattern of growth; therefore, “active observation” is recommended. However, about 10% of cases are referred to as high-risk hemangiomas (Figure 1) because of their size, character, or location and are in need of immediate intervention. Indications for different kinds of treatment are shown in Figure 2 [20].

### 3.1. Propranolol

Propranolol is a nonselective beta-adrenergic receptor antagonist approved by the Food and Drug Administration (FDA) in 2014 for the treatment of IH. It is administered in the form of an oral solution. The FDA recommends beginning the therapy with a dose of 0.6 mg/kg twice daily, then increasing the dose to 1.1 mg/kg twice daily after one week and to 1,7 mg/kg twice daily after two weeks. Leaute-Labreze et al. and Baselga et al. recommend starting the treatment with 1 mg/kg/day in two doses in the first week, 2 mg/kg/day in the second week, and 3 mg/kg/day in the following week [21,22]. A higher dose of up to 3 mg/kg/day has been used in Alder Hey Hospital and has been shown to be effective and well tolerated [23]. However, some authors reserve the dose of 3 mg/kg/day only for resistant IH [24]. The treatment should be started in the proliferative phase of IH growth, even in neonatal age, and maintained for 6 months. The authors of a prospective study concerning the individualized dosage of propranolol in the treatment of infantile hemangiomas suggest starting the treatment with the low dose of 1–1.5 mg/kg/day, and if the patient responds well, the therapy should be continued at the same dose to decrease the risk of potential adverse effects. Patients who do not respond at all to the lower dose after 1 month of therapy are unlikely to benefit from the propranolol treatment overall [25]. It is important to readjust the dose with the increase of the child’s weight.

The child’s caregivers should be instructed to administer propranolol orally after a meal in order to reduce the risk of hypoglycemia. The novel methods of administration of propranolol include propranolol-loaded mesoporous silica nanoparticles and continuous delivery from liposomes-in-microspheres. Despite promising results in studies of tumor inhibition in mice, the safety of this type of therapy is yet to be established [26,27].

#### 3.1.1. Mechanism of Action

It is believed that propranolol acts on IH in multiple paths, and different results of action are achieved progressively during the treatment. The initial response, visible as color fading and reduced consistency, is a result of vasoconstriction, which reduces the blood flow through the IH vessels. The proposed mechanism of early response is the inhibition of beta-2 receptors by propranolol, which blocks the vasodilatation of the vessels mediated by adrenaline through the activation of endothelial nitric oxide (NO) synthase [28]. The mid-term effect of arresting the growth and progression of IH might be associated with reducing the levels of cytokines involved in its growth. HIF-1-alpha, which has been shown to be upregulated in the proliferative phase of IH, increases the levels of VEGF and MMPs produced by tumor cells [29,30,31]. Propranolol inhibits the action of norepinephrine at the cell level, which physiologically stimulates the production of HIF-1-alpha, causing the indirect reduction of VEGF and MMPs [32]. Natural involution might be the result of increased local oxygen supply due to the formation of new vessels in the proliferative phase, causing the decrease of HIF-1-alpha and subsequently VEGF and MMPs [28,33]. The long-term effect of reducing the size and color along with the usual leftover fibrofatty tissue is probably achieved by promoting the differentiation of IH stem cells into adipocytes instead of pericytes and endothelial cells, as well as the induction of apoptosis in existing endothelial cells [28,34].

Another potential mechanism of action is the reduction of renin induced by beta-blockers. It has been shown that the components of the RAA axis are present on IH-derived cells, and their levels are elevated in the sera of patients with IH compared to the control group [17].

Sasaki et al. studied human endothelial stem cells derived from IH and a murine model of infantile hemangioma treated with different propranolol isomers: R-propranolol (inactive towards beta-receptors) and S-propranolol (active towards beta-receptors). Interestingly, the results showed a better effectiveness of R-propranolol in downregulation of the Angiopoietin-like 4 (ANGPTL4) gene expression which is present in human IH. Moreover, their previous study concerning cytokines in IH showed that propranolol reduces VEGF in children but not the angiopoietin-2 (ANG2) levels in their serum. They hypothesize that the action of propranolol might be beta-blockade independent (although several beta-blockers have shown their effectiveness), and ANG2 might cause apoptosis of endothelial IH cells in the absence of VEGF and ANGPLT4 [35].

Moisan et al. identified in IH a previously unrecognized perivascular layer composed of telocytes (TCs) which express Aquaporin-1 (AQP1) [14]. TCs are dendritic cells and are suggested to take part in intercellular communication, among other functions [36]. The authors conclude that propranolol may act on IH upon the beta-adrenergic-2 receptor (ADRB2), which triggers the same pathway as the downregulation of AQP1 [14].

#### 3.1.2. Recurrence and Residual Lesions after Propranolol Treatment

The known risk factors of IH recurrence are female sex, tumor size over 50 cm^3^, tumor location on head and neck, deep or mixed type hemangioma, and early treatment withdrawal (<9 months) [37]. Kagami et al. suggest that patients with IH should be monitored for up to 6 months after the cessation of treatment [38]. In a follow-up study of the outcomes of propranolol treatment, in a group of 80 patients, residual lesions were found in 91.2% (73/80). Most of them presented as telangiectasias, fibrofatty tissue, or erythema. The mixed hemangiomas had the highest residual rate of 94.1%, but the superficial ones left a significant or severe residual lesion in 83.4% of cases [39]. Chang et al. showed different expressions of 22 miRNAs in 18 recurrent patients compared to 20 nonrecurrent patients. The potential target genes of these miRNAs are related to vascular remodeling; cell signaling, growth, adhesion, and differentiation; and basement membrane metabolism [40]. 

#### 3.1.3. Visceral Hemangiomas

One of the indications for propranolol treatment is visceral hemangioma, with a mortality rate of 81% if left untreated [41]. The most common localizations are on the liver and the lungs, where propranolol treatment has shown a satisfactory response [42,43].

#### 3.1.4. Side Effects

Up to 2% of treated patients develop mild side effects, most commonly sleep disturbances and irritability. Other potentially serious side effects include bronchospasm, bradycardia, hypotension, and hypoglycemia. Ceasing the therapy and restarting after the disappearance of symptoms is found to be well-tolerated without any further complications [1]. A meta-analysis performed by Yang et al. showed no significant difference in the occurrence of side effects and relapses between oral propranolol and other therapies [6].

### 3.2. Other Beta-Blockers

#### 3.2.1. Atenolol

A case series of 46 infants showed a great response to oral atenolol in doses ranging from 1 to 3 mg/kg administered once a day. Atenolol is a selective beta-1 blocker with no effect on beta-2 receptors, thus no risk of bronchospasm or hypoglycemia and potentially dangerous side effects of using propranolol. A decreased frequency of sleep disturbances is also observed during atenolol treatment when compared to propranolol, and this fact may be associated with a reduced passing of the blood–brain barrier caused by its hydrophilic properties. However, a study by Laurens et al. showed that both lipophilic propranolol and hydrophilic atenolol are able to modulate NO release in the hypothalamus [44]. It is worth mentioning that no recurrence after the end of the treatment was observed, and a mean time of 16,4 days was required to close the wounds of ulcerated hemangiomas. The most common side effect was mild transient diarrhea affecting almost one-quarter of patients [45]. Another case report showed similar results concerning less-frequent sleep disturbances than during propranolol treatment [46].

#### 3.2.2. Timolol

Timolol is widely used as a topical treatment of infantile hemangiomas. In the first systematic review and meta-analysis of the treatment of IH with topical timolol, the authors concluded that topical timolol is safe and effective for the treatment of small, uncomplicated, and superficial hemangiomas. However, the authors stress the need for a randomized control study in order to further establish its role in the guidelines of IH treatment [47]. Wu et al. propose topical timolol as a first-line treatment for superficial hemangiomas as it showed similar efficacy to propranolol with fewer systemic adverse events [48]. Another meta-analysis proved that timolol showed a significantly better response rate and fewer adverse effects in comparison to laser treatment, placebo, and the control group. There were no significant differences in comparison to propranolol treatment [49]. On the contrary, Ying et al. showed in a prospective study that topical timolol had a worse visual effect than PDL laser treatment [50]. A recent randomized clinical trial confirmed the safety of 0.5% timolol maleate solution but showed no significant differences in IH resolution in comparison to placebo when administered in the early proliferative stage [51]. Topical timolol was also reported to be effective in the treatment of an iris hemangioma [52].

He et al. analyzed ultrasonographic measurements at the first visit and after 1 month of treatment with 0.5% topical timolol. They suggest that arterial diameter (AD), venous diameter (VD), resistance index (RI), pulsatility index (PI), and peak arterial systolic velocity (PASV) might be used as treatment response predictors. They observed the highest regression rates in hemangiomas of superficial type located on the torso with the treatment initiation at 5–6 months. However, because of a lack of a control group, it was impossible to differentiate the effects of timolol treatment from the natural involution of IH [53].

It has been reported in a prospective cohort study that timolol applied topically absorbs into the bloodstream, and the location of IH may have an impact on the level of absorption. Besides systemic absorption, timolol appears not to be associated with severe side effects [54].

Almebayadh et al. presented two cases of the successful treatment of ulcerated hemangiomas treated with brimonidine 0.2%-timolol 0.5% cream (a combination of a selective alpha-2-adrenergic agonist and a non-selective beta-blocker), although it is not recommended to combine the treatment with oral propranolol as the risk of severe side effects increases [55,56].

On the other hand, Mannschreck et al. showed a good therapeutic effect of propranolol followed by topical timolol compared to propranolol therapy alone. The combined therapy had a shorter course, the patients did not present any side effects, and no recurrence was observed. These authors suggest that the use of topical timolol with systemic propranolol therapy may minimize the potential side effects of the treatment by shortening the overall therapy course [57]. A meta-analysis performed by Qiao et al. also confirmed a better response rate of the combined treatment in comparison to propranolol or timolol treatment alone [58]. In one study, oral propranolol combined with topical timolol was found to have an overall response of 100% in the treatment of compound IF. The authors suggest, as do some other researchers, that the treatment should be initiated as early as possible, in the proliferative phase of the IH, in children younger than 12 months [59]. 

### 3.3. Other Drugs

In case of a lack of response to the propranolol treatment, other therapeutic options may be offered to the patient, including intralesional pingyangmycin, intravenous vincristine, and oral steroids. Zhang et al. treated 19 patients with poor response to propranolol with intralesional pingyangmycin (*n* = 11) and intravenous vincristine (*n* = 8). In 17 patients, they achieved good therapeutic effect, and only 2 needed surgical resection [60]. Due to possible severe side effects—pulmonary fibrosis for pingyangmycin and neurotoxicity for vincristine—these drugs remain as optional treatment for infantile hemangioma.

### 3.4. Laser Treatment

Since the development of the theory of selective photothermolysis, lasers have been widely used for treating cutaneous lesions, including infantile hemangiomas. Laser stands for light amplification by stimulated emission of radiation, and the technology uses an active medium that delivers atoms to generate electromagnetic radiation. The type of medium usually determines the name of the laser. The most common laser used for the treatment of infantile hemangiomas is the pulsed dye laser (PDL).

#### 3.4.1. Mechanism of Action

The atoms from an active medium absorb the energy (in the medical field, the source of energy is usually electric current), which results in their achievement of a high-energy state. The energy becomes coherent and collimated as it reflects between the mirrors in the chamber. One of the mirrors allows for the energy to escape as a beam of laser radiation due to its partially reflective properties. The light reaching the skin might be reflected, absorbed, scattered, or transmitted. A good therapeutic effect is obtained when most of the light is absorbed by targeted molecules, called chromophores, in IH oxyhemoglobin. PDL emits light at wavelengths of 585 and 595 nm, which targets the second oxyhemoglobin peak. In order to minimize the damage to surrounding tissues, the wavelength should be as close as possible to the targeted one. Another important factor is the duration of the laser pulse. It is determined by the thermal relaxation time, which is the time needed for the tissue to come back to its proper temperature after heating with a laser beam. Heating for longer than the thermal relaxation time of the targeted chromophore results in collateral damage. Longer pulse duration (10 ms) has fewer side effects (e.g., erythema, edema, purpura) compared to shorter pulse durations (1.5–3 ms) [61]. The efficacy of PDL also varies depending on the localization of the infantile hemangioma. In a retrospective study among a Chinese population, lesions on the trunk and extremities showed the highest excellent response rates, 71.43% and 68.85%, respectively. On the face, 100% of excellent responses were observed on the upper lip and the temporal area, 75% on the nose, 71.43% on the eyelid, 66.67% on the forehead, and only 57.14% on the cheek [62].

It has been shown that PDL treatment significantly reduces VEGF levels in the sera of patients, similarly to propranolol. Interestingly, the level of VEGF after three laser treatments decreased nearly by half, while the mean lesion size did not differ significantly. Moreover, a downregulation of VEGF mRNA expression in human umbilical vein endothelial cells (HUVECs) and an increase in apoptosis rate in vitro have also been observed. The cellular changes on scanning electron microscopy include condensation and fragmentation of the nucleus and swollen mitochondria [63].

Laser treatment is not recommended during the proliferative phase of IH growth [20,64,65] due to a lack of effectiveness and the risk of potential complications [66]. However, Zhang et al. showed that the use of PDL in neonates requires less energy and reduces recovery time between sessions compared to non-neonates [67]. Laser treatment plays a significant role in treating residual lesions, such as telangiectasia [64].

#### 3.4.2. Laser versus Observation

When compared to an observation group, a laser-treated group showed better cosmetic results with more color fading and arresting of the proliferative phase of growth more rapidly [64]. A study reviewing 432 case reports of IH after pharmacological and dye-laser treatment reported the lowest incidence of residual lesions after laser treatment compared to untreated patients [68].

#### 3.4.3. Neodymium-Doped Yttrium Aluminum Garnet (Nd:YAG) Laser

The Nd:YAG laser is the second most common type of laser used to treat vascular lesions. It emits the beam of light at mid-infrared wavelengths (1064 nm), which corresponds to an additional infrared absorption peak of oxyhemoglobin at around 1000 nm. As it penetrates deeper (5–6 mm) than PDL (0.75–1 mm), it is indicated in the treatment of deep or mixed hemangiomas, especially with the combination of both types of lasers [69,70]. The main disadvantage is the pain experienced during the laser session, which may require the use of general or local anesthesia [70,71,72].

### 3.5. Combined Treatment

Parallel treatment of IH with systemic propranolol and combined Nd:YAG/PDL laser therapy was found to be safe and effective. It did not seem to reduce the time of the treatment, but fewer side effects were noted [73]. On the contrary, a recent study by Sugimoto et al. showed that combined propranolol and PDL therapy might reduce the duration of the propranolol administration [74].

In a double-blinded randomized controlled trial, Asillan et al. found that combined PDL laser and timolol gel were more effective than PDL alone, but only if started after 3 months of age [75]. Chen et al. performed a randomized controlled trial among patients with ulcerated infantile hemangioma treated with topical timolol or PDL. No statistically significant difference was observed between the two types of treatment, although the authors mention that topical timolol is less expensive, which should also be taken into consideration in light of the economic aspect of healthcare [76].

Although it is not currently common, sclerotherapy still remains an option in the therapy of vascular lesions, especially combined with other forms of treatment. Lauromacrogol with PDL and Nd:YAG laser sessions have a good therapeutic effect and may serve as an alternative for patients who cannot tolerate other treatment. A possible side effect of sclerotherapy is ulceration. Thus it is not recommended in therapy of IH localized on the face [77,78].

## 4. Conclusions

Infantile hemangiomas, although having benign character, in some situations may cause life-threatening complications, permanent disfigurement, and functional impairment. In such cases, treatment is recommended over an active observation strategy. For high-risk infantile hemangiomas (Figure 1), systemic propranolol should serve as the first-line therapy. It is recommended to start the treatment early, in the proliferative phase of growth of IH. Topical timolol might be offered to patients with small infantile hemangiomas and a low risk of complications. Laser therapy should be considered both as a single treatment option as well as a complimentary treatment of residual lesions. Primary indication for laser therapy is superficial hemangioma. In case of insufficient response to standard treatment, other options include sclerotherapy, vincristine, steroids, and surgical resection.

## Data Availability

Not applicable.
